# The Footprint of Continental-Scale Ocean Currents on the Biogeography of Seaweeds

**DOI:** 10.1371/journal.pone.0080168

**Published:** 2013-11-08

**Authors:** Thomas Wernberg, Mads S. Thomsen, Sean D. Connell, Bayden D. Russell, Jonathan M. Waters, Giuseppe C. Zuccarello, Gerald T. Kraft, Craig Sanderson, John A. West, Carlos F. D. Gurgel

**Affiliations:** 1 UWA Oceans Institute & School of Plant Biology, University of Western Australia, Perth, Western Australia, Australia; 2 Marine Ecology Research Group, School of Biological Sciences, University of Canterbury, Christchurch, New Zealand; 3 Environment Institute & Southern Seas Ecology Laboratories, School of Earth and Environmental Sciences, the University of Adelaide, Adelaide, South Australia, Australia; 4 Department of Zoology, University of Otago, Dunedin, New Zealand; 5 School of Biological Sciences, Victoria University of Wellington, Wellington, New Zealand; 6 School of Botany, University of Melbourne, Melbourne, Victoria, Australia; 7 School of Zoology, University of Tasmania, Hobart, Tasmania, Australia; 8 South Australian State Herbarium, Science Resource Centre, Department of Environment & Natural Resources, Adelaide, South Australia, Australia; 9 South Australian Research and Development Institute, Aquatic Sciences, Adelaide, South Australia, Australia;; University of New South Wales, Australia

## Abstract

Explaining spatial patterns of biological organisation remains a central challenge for biogeographic studies. In marine systems, large-scale ocean currents can modify broad-scale biological patterns by simultaneously connecting environmental (e.g. temperature, salinity and nutrients) and biological (e.g. amounts and types of dispersed propagules) properties of adjacent and distant regions. For example, steep environmental gradients and highly variable, disrupted flow should lead to heterogeneity in regional communities and high species turnover. In this study, we investigated the possible imprint of the Leeuwin (LC) and East Australia (EAC) Currents on seaweed communities across ~7,000 km of coastline in temperate Australia. These currents flow poleward along the west and east coasts of Australia, respectively, but have markedly different characteristics. We tested the hypothesis that, regional seaweed communities show serial change in the direction of current flow and that, because the LC is characterised by a weaker temperature gradient and more un-interrupted along-shore flow compared to the EAC, then coasts influenced by the LC have less variable seaweed communities and lower species turnover across regions than the EAC. This hypothesis was supported. We suggest that this pattern is likely caused by a combination of seaweed temperature tolerances and current-driven dispersal. In conclusion, our findings support the idea that the characteristics of continental-scale currents can influence regional community organisation, and that the coupling of ocean currents and marine biological structure is a general feature that transcends taxa and spatial scales.

## Introduction

Understanding how regional-scale processes contribute to geographic structuring of biodiversity is challenging because many possible environmental drivers often correlate across broad spatial scales. A starting point is the recognition that increasing geographical distances between observations reveals increasing dissimilarities among biological communities, a phenomenon reflected in distance decay curves [1]. In addition to documenting biogeographic patterns, distance decay curves also provide insights into the nature of the processes which underpin these patterns, through variation in the rates of distance decay of community similarity – species turnover - between geographical regions [1,2]. 

Marine biogeographers have traditionally held that broad-scale patterns of species distributions result primarily from species-specific responses (e.g., larval biology, thermal physiology) to environmental clines such as those associated with continental-scale current systems [3,4]. Temperature, in particular, has been invoked to explain biogeographic patterns for centuries [5,6]. These early accounts are supported by contemporary quantitative studies which show how temperature can be a strong predictor of spatial changes in species composition and biodiversity [7-10]. Whilst temperature clearly is a powerful driver of biogeographic patterns in marine systems, the interpretation of patterns in relation to alternate drivers that might by superimposed onto the temperature signal have received comparatively less attention. Recently, however, marine biogeographers have drawn attention to the role of physical connectivity mediated by current flow and water movement, even across large distances [11-14].

Marine systems differ from terrestrial systems in typically being more ‘open’, with fewer physical barriers to biological exchange between regions [15]. Community structures are therefore often strongly influenced by the passive dispersal of microscopic propagules (spores, gametes, larvae) and macroscopic drifting or swimming organisms, from adjacent areas, with delivery mediated by water movement [e.g., 16,17]. Major ocean currents can provide a link between neighbouring (kilometres) and distant (100’s kilometres) habitats. Model calculations have shown that, in some places, flow patterns alone can explain species range-limits [13]. Evidence on how oceanic flow structures such as up-welling, down-welling, eddies and along-shore water transport influence the recruitment and population structure of marine invertebrates across spatial scales up to 100’s of kilometres have gained increasing support [16-18]. Recent analyses of genetic connectivity in sea urchins [19] and seaweeds [12,20] provide further evidence of significant correlations between water movement, oceanographic connectivity, species distributions, and gene-flow. However, such links between oceanographic connectivity and biological structures should also be evident across larger spatial scales (i.e. continents) and higher levels of biological organisation (i.e. communities within entire seaweed floras).

**Figure 1:**
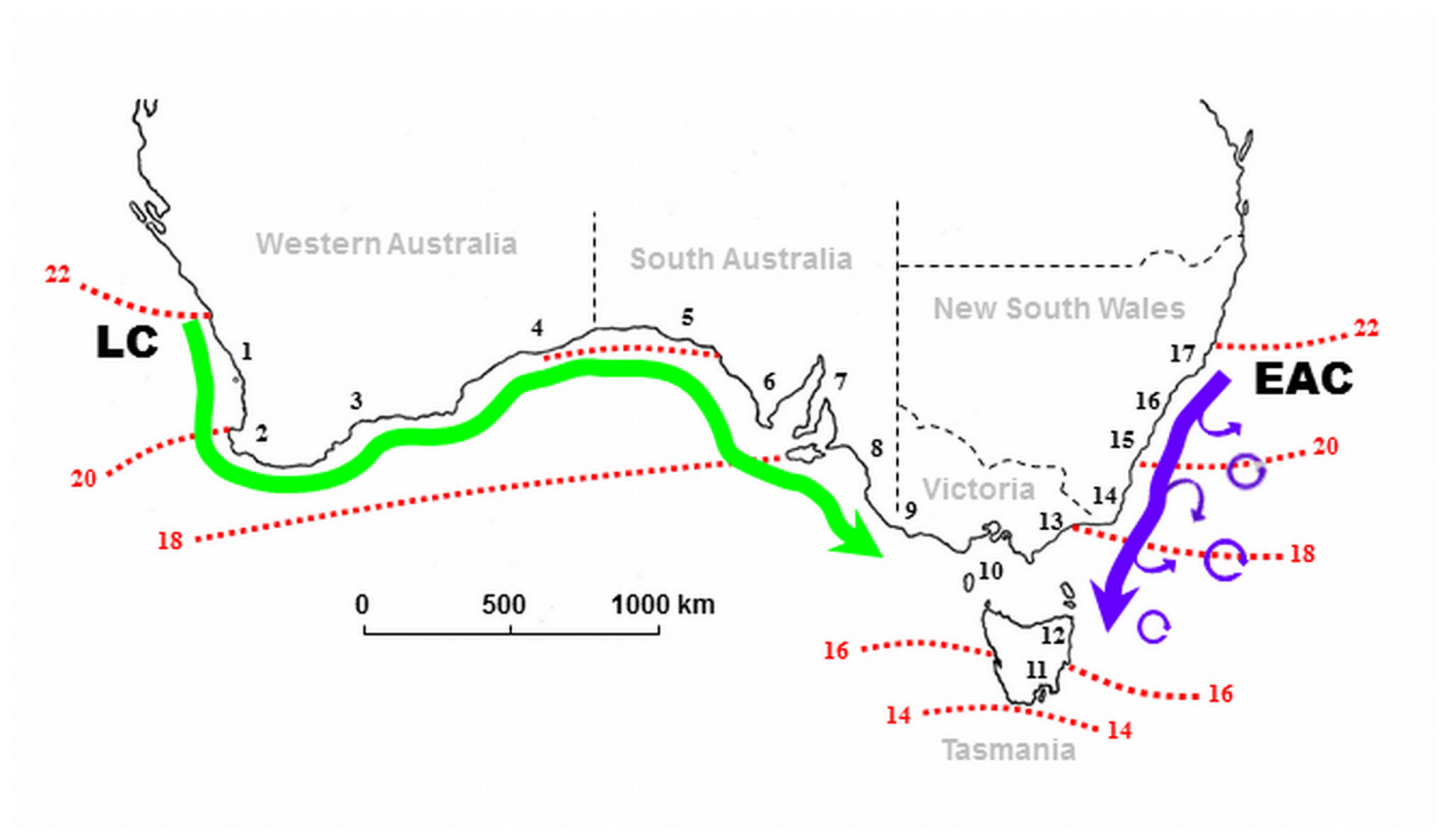
Map of temperate Australia indicating the flow of the two main surface currents. The Leeuwin Current (LC = green arrow) has a relatively uninterrupted and highly connective flow along the west and south coast whereas the East Australia Current (EAC = blue arrow) has a more heterogeneous flow, with eddies frequently spinning offshore, along the east coast [after 24]. Numbers 1-17 refer to bioregions based on the National Marine Bioregionalisation of Australia [25, see Table S1]. Red dotted lines indicate summer (January, °C) isotherms [after 69]. Temperature profiles across the currents are relatively similar, despite the LC flowing across almost three times the geographic distance as the EAC.

The coastal waters of temperate Australia are dominated by two major boundary currents: the Leeuwin Current (LC, [21]) and the East Australia Current (EAC, [22]). Both currents are relatively old in geological terms, warm and both flow pole-wards across approximately the same latitudes on either side of the Australian continent (Figure 1). Whilst temperature ranges are relatively similar between the LC and the EAC, the LC covers >3 times the geographical distance of the EAC (Figure 1), implying that the spatial temperature gradient is much steeper along the EAC. Such a steep environmental gradient should be reflected in narrow distribution ranges of individual species and thereby relatively high turnover of species between communities [1]. In addition, the two currents also differ in flow structure. The EAC is a stronger current which is periodically disrupted by eddy formation where pockets of water are spinning offshore (see Figure 1). In contrast, the LC is a weaker but relatively undisrupted and unidirectional current [12,21,23,24]. Connectivity modeling has shown that these differences in current characteristics can have important consequences for local retention, off-shore advection and cross-shore transport of marine organisms [24]. 

Here we use an extensive database of seaweed herbarium records for biogeographic analyses aimed at identifying and understanding continental-scale patterns in seaweed community organisation. Given the predominant direction of dispersal and connectivity we hypothesised that seaweed communities would change serially across regions, in the direction of current flow. Moreover, given that the LC has a weaker temperature gradient and more uniform uninterrupted along-shore flow, compared to the EAC’s steep temperature gradient and disrupted flow, we hypothesised that seaweed communities within the LC would show less spatial structure and lower species turnover compared to seaweed communities within the EAC.

## Methods

This study encompassed the entire temperate coastline of Australia, which is swept by the LC and the EAC (Figure 1). Our spatial units of analysis were 17 ‘bioregions’, 10 associated with the LC and 7 associated with the EAC (Figure 1, Table S1). These mesoscale bioregions were derived from the National Marine Bioregionalisation of Australia (IMCRA) [25], designed as marine management units based on geomorphology, oceanography and biological communities. We used these IMCRA regions as sampling units because they represent units of community characterisation independent of spatial extent of coast. Seaweed communities for each region were constructed by compiling species presences from records of seaweeds (marine macroalgae) lodged in the Australian Virtual Herbarium [26] as of 14 September 2009; experienced seaweed taxonomists (G. T. Kraft and C. F. D. Gurgel) inspected all downloaded electronic records [27] and carried out a detailed taxonomic revision, updating and standardizing nomenclature according to Algaebase, an online resource for seaweed taxonomy [28]. While there are many possible challenges associated with the analysis and interpretation of herbarium data, they represent a viable and comprehensive source of information for continental-scale analyses; the very large size of our units of analysis reduce the limitations associated with false absences, uneven recording effort and the dynamic nature of species distributions [29]. For example, recent changes to the distribution of seaweeds have been recorded along all coastlines in Australia [30-33], but at distances substantially smaller than our bioregion-scale of analysis (Table S1).

We used the Bray-Curtis similarity index based on presence-absences to compare community structure among bioregions within current systems. A series of multivariate analyses were performed in PRIMER 6.1.10 & PERMANOVA+ for PRIMER [34 for technical details,tests refer to routines in this program, see 35]. Principal Coordinates Ordination (PCO) was used to visualise patterns of community similarity among regions, as this unconstrained metric ordination, show optimal inter-relationships between data in a non-preconceived way. Multivariate analysis of variance by permutation (PERMANOVA) and Permutational analysis of multivariate dispersion (PERMDISP) tested if seaweed communities were different, and had different levels of variation, between the LC and EAC. We also tested the strength of serial rank-correlation in community structure between successive bioregions for each of the two current systems (RELATE, using the default model matrix for serial change). Finally, we plotted community similarity as a function of spatial separation distance (measured as linear coastal distances from the centre of each region) and calculated species turnover as the slope of log-linear regression lines. Analysis of covariance (ANCOVA) tested if turnover differed between the two current systems (small slope = adjacent communities are similar = low species turnover).

## Results

There were a total of 80,188 records of seaweeds in AVH for the 17 bioregions, with an average (± SE) of 4,717 ± 1,152 records per region. There was relatively similar sampling effort (= number of herbarium specimens) between current systems, with 9.1 ± 1.7 (n = 10 regions) and 4.8 ± 1.1 (n = 7 regions) specimens km^-1^ coastline within the LC and the EAC systems, respectively (*t*-test for unequal variances, *t*_*10,7*_ = 2.04, *P* = 0.061, see also Table S1). This effort resulted in a total of 1,499 recorded species with an average of 490 ± 54 species per region and similar species densities of 0.96 ± 0.19 and 0.88 ± 0.12 species per km of coastline within the LC and the EAC, respectively (*t*-test for unequal variances, *t*_*10,7*_ = 0.35, *P* = 0.733).

**Figure 2:**
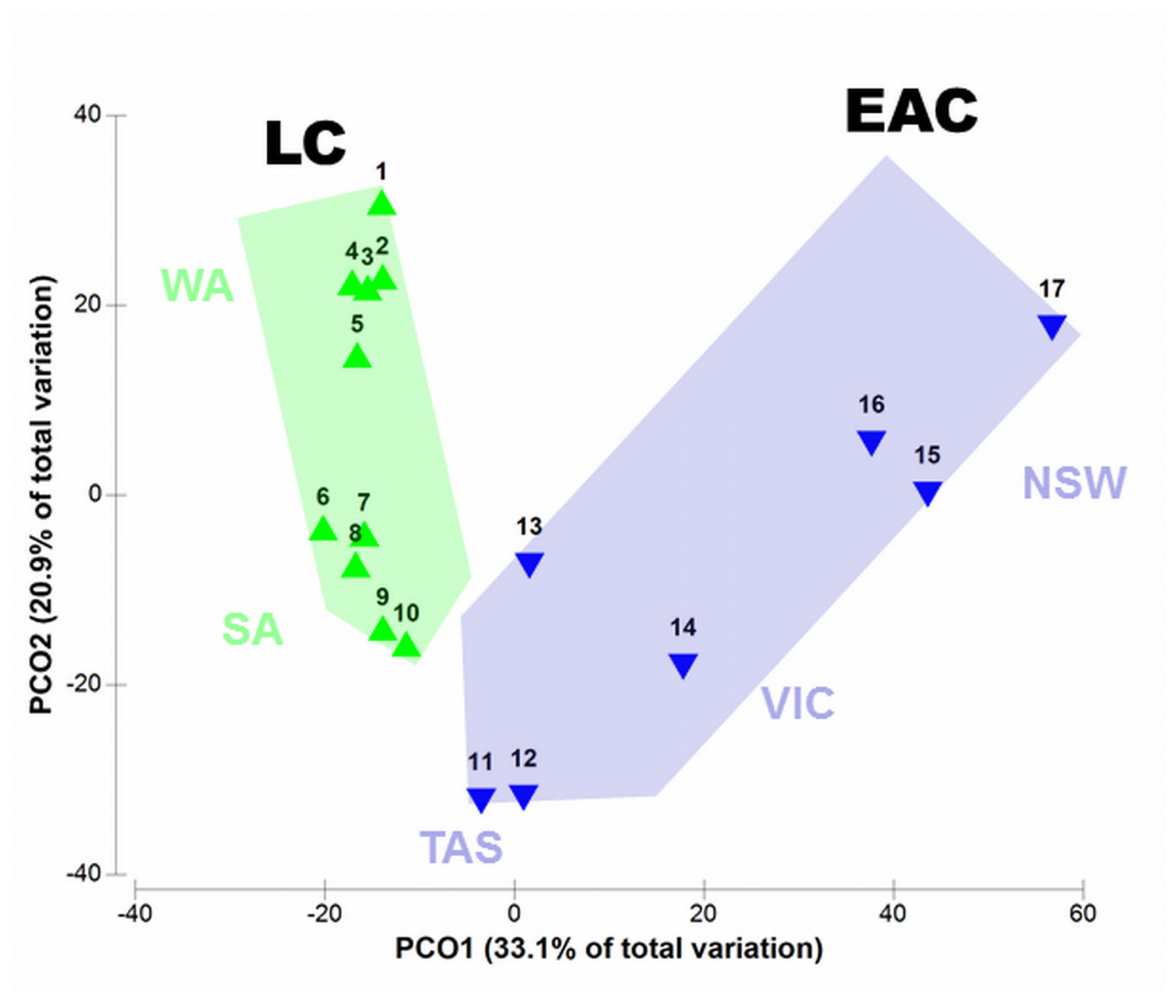
Principal Component Ordination (PCO) of seaweed community structure from bioregions swept by the Leeuwin Current (LC = green triangles) and the East Australia Current (EAC = blue triangles). See Figure 1 for spatial arrangement of regions. The green and blue arrows indicate the current systems super-imposed onto the ordination to illustrate the magnitude of floristic change across the current system (length of arrow) and the variation in community structure among regions within the system (width of arrow). There was significant sequential change in community structure along both current systems.

Ordination of seaweed communities placed the 17 bioregions into two groups, clearly separating regions influenced by the LC from those influenced by the EAC (Figure 2). Overall, the LC and EAC groups were significantly different (*F*_*1,15*_ = 5.2, *P* = 0.001), with highly distinct assemblages between the northern, subtropical regions in Western Australia and New South Wales, converging in the cool-temperate regions of Tasmania (see also Table S2). Moreover, the ordination show the contrasting patterns of serial correlation between regions within the two current systems, with ‘short-narrow’ relations between LC regions and ‘long-wide’ relations between EAC regions (Figure 2). In both current systems, the serial change from region to region was significant (*ρ*_*LC*_ = -0.611, *ρ*_*EAC*_ = -0.762, *P* < 0.004), but the multivariate dispersion between regions was significantly smaller within the LC (29.0 ± 2.0, ‘narrow’ arrow) than the EAC (41.0 ± 2.7, ‘wide’ arrow) (*F*_*1,13*_ = 13.2, P = 0.003).

**Figure 3:**
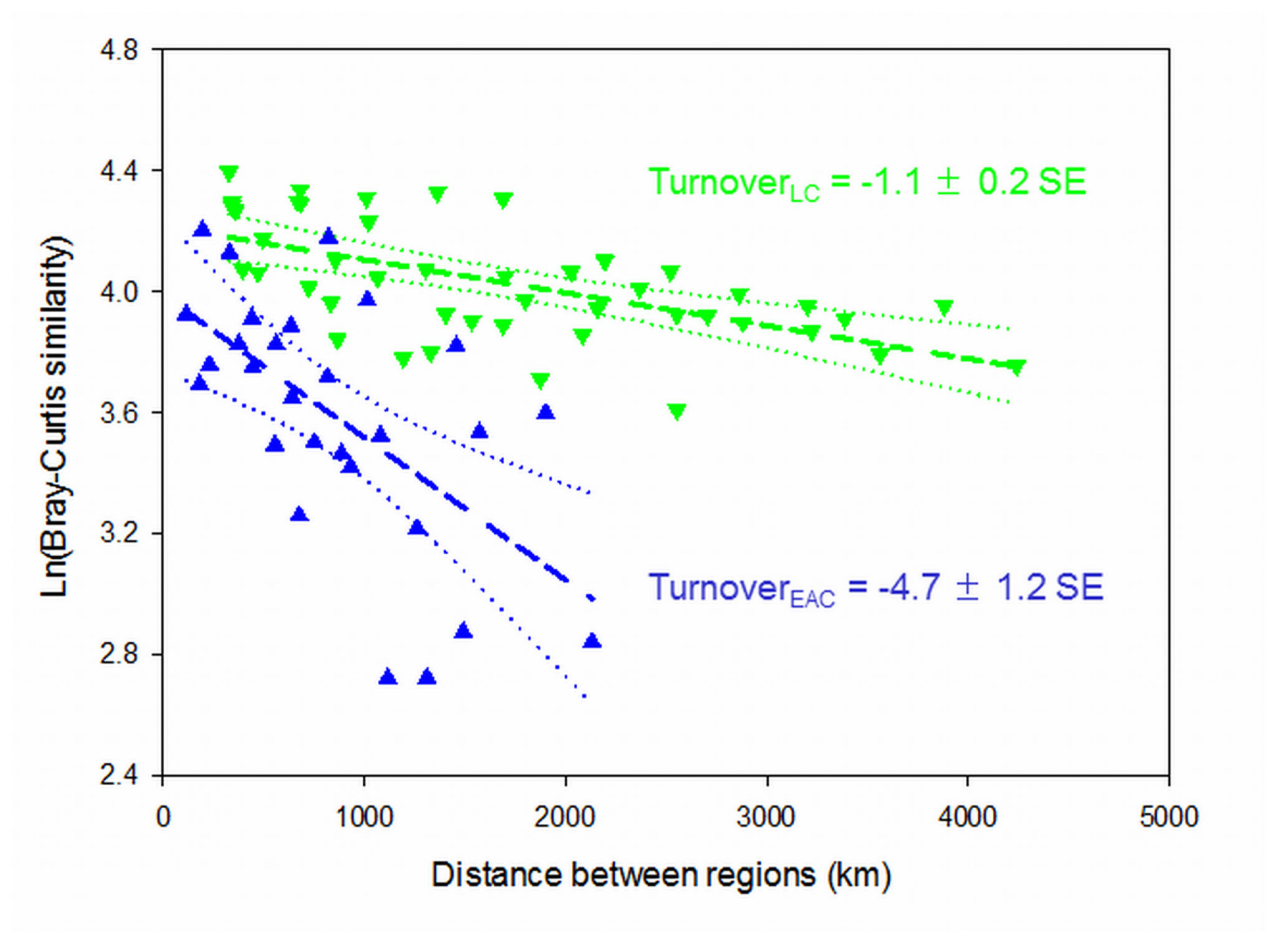
Species turnover between bioregions swept by the Leeuwin current (LC = green triangles) and the East Australian current (EAC = blue triangles). Dashed lines represent linear regression of community similarity against coastal distance between regions. Dotted lines are 95% confidence limits around the regression lines. Turnover (slope × 10^4^) within the LC system is significantly lower than within the EAC system. At the same time, regional seaweed communities within the LC were more similar (similarity values were higher), and associated with less region-to-region variability (less spread around the regression) than within the EAC.

Log-linear regression analyses revealed highly significant negative relationships between spatial separation distance and similarity in seaweed community structure within both current systems (Figure 3, r ^*2*^_*LC*_ = 0.36, *r* ^*2*^_*EAC*_ = 0.36, *P* < 0.001). Species turnover (slope of the regression lines) was significantly different between the two current system (*F*_*1,69*_ = 14.87, *P* = 0.0002) and > 4 times greater within the EAC than the LC (Figure 3).

## Discussion

Understanding the processes that underpin broad-scale patterns in species distributions is becoming increasingly important because of the pressing need to integrate information across broader spatial scales to resolve issues of human impacts (e.g., climate change, fishing, trophic cascades, eutrophication) [36]. The present study shows a strong imprint of continental-scale ocean currents on the biological community structure of seaweeds across temperate Australia, with high community variability and rapid regional species turnover coinciding with large-scale heterogeneity of current patterns and a strong temperature gradient. Our study thus adds to the increasing support for the idea that broad-scale patterns of species distribution and community structure can be mediated by a combination of well-known temperature gradients and less-studied flow patterns [11,13].

It is well-established that high species turnover (rates of decay in community similarity) is associated with steep temperature (and other environmental) gradients in terrestrial and freshwater systems [1,37-39], but fewer marine studies have quantified change in community structure across large spatial scales [but see 40 for an example]. In marine systems, flow structure as a driver of biogeographic patterns has previously been associated with regional differences in abundance, recruitment [16-18,41] and genetic structure [19,42] of invertebrates and a single seaweed species [12]; our results extend these findings to encompass continental-scale patterns in entire seaweed communities. Collectively, these studies demonstrate how the physical structure of ocean circulation act as footprints that impart structure transcending taxa and spatial scales.

### Influence of temperature

Marine organisms tolerate a limited range of temperatures for their survival, growth and reproduction. Temperature variation is therefore a fundamental physiological constraint to global-scale distribution of seaweeds [8,43]. This well-established rule is possibly amplified by the present day distribution of coastlines that generally orientate north to south (e.g., E/W Atlantic Ocean, E/W Pacific Oceans, E/W) so that most seaweed communities live within steep spatial temperature gradients. Hence, most seaweed biogeography has been interpreted as temperature driven [8,44-48]. Still, the causal factors underlying marine biogeographic patterns may not always be so straightforward [49,50], and this difficulty has been particularly evident in southern Australia where temperature gradients are weak [e.g., 51]. As oceanographic currents often drive changes in temperature regimes, marine biogeographers have often interpreted associated biogeographic transitions in terms of temperature [50], to the exclusion of alternative explanations such as those that involve flow connectivity alone [13]. For example, Schils & Wilson [7] attributed very sharp floristic changes in the Arabian Sea to temperature, but did not consider the potential contribution from effects of oceanographic circulation. To date, most biogeographic seaweed studies have focused on how large scale latitude-temperature patterns influence species identities and species richness [43,47,48,52], rather than how temperature gradients modify species turnover. We documented patterns in seaweed communities likely to reflect continental-scale temperature gradients with strong serial community transitions within both of the major current systems influencing temperate Australia. Moreover, species turnover was higher within the EAC (steep temperature gradient) than within the LC (flat temperature gradient). Nevertheless, temperature might not account for all region-to-region seaweed species turnover or the level of variability among regions. Indeed, a study of species turnover in marine invertebrates reported no direct relationships with temperature, although they did not present any alternative explanation for the biogeographic patterns detected [51]. We propose that oceanographic transport of microscopic propagules and drifting reproductive fragments could also be a key contributing structuring process in Australia’s seaweed biogeography [12,24,53], and that this model might have broad biological relevance beyond Australia.

### Influence of flow

Oceanographically mediated dispersal has been considered to shape spatial patterns of community similarity of coastal fish and invertebrates [11,18]. Ocean circulation may directly link with patterns of propagule dispersal, particularly around biogeographic disjunctions [13]. Given that both the LC and EAC flow southward and that seaweeds are passive dispersers that rely strongly on transport of drifting adult thalli for long-range dispersal (microscopic propagules typically settle metres from the parent plant) [43,54-58], it seems plausible that different serial correlation in community structure and species turnover are, at least partly, explained by flow. Moreover, off-shore currents have previously been shown to be a barrier to dispersal [24,41,59] and could contribute to differences in floristic heterogeneity among regions within the LC and EAC, with drifting propagules more likely to be transported away from the coastline within the EAC [24].

The co-variation between flow patterns and temperature gradients prevent an unambiguous separation of their influences on broad-scale patterns in seaweed community structure. However, comparative studies, testing the physiological and ecological performance of species and populations across latitudinal temperature regimes [60,61,62] might shed light on the extent to which the patterns are primarily temperature or flow driven. Such comparisons will be particularly important in terms of understanding the extent to which impending ocean warming might cause shifts in species distribution, relative to other climate stressors in this system [36,63,64].

### Influence of other factors

Temperature gradients and off-shore flow might not be the only mechanisms that contribute to the extant biogeographic patterns. Historical factors, such as the relative stability of the current systems and lack of large-scale disruptions (e.g., glaciation) over millions of years, which contrasts most other temperate regions in the world, are likely to have had a major influence on the evolution and biogeography of the temperate seaweed flora of Australia [46,65]. However, while these factors might explain the mega-richness of the flora and lack of major disjunctions, they are less likely to be a cause of regional patterns in species turnover and community structure. Additional environmental heterogeneity unrelated to ocean currents (e.g., estuaries), human activities [66,67], or biological interactions such as overgrazing by sea urchins [60] could also add to community heterogeneity. However, most of these features operate at spatial scales smaller than our units of analyses and over relatively short time-scales. Differences in niche breadths or dispersal capabilities of seaweeds within the LC and EAC would also influence conclusions about species turnover [1]. However, there are no reasons to suspect that such systematic differences should exist between current systems. Finally, the EAC is characterized by faster current speed and transports a greater volume of water compared to LC [68]. However, these flow characteristics should decrease species turnover along the east coast (opposite to our findings) and are therefore unlikely explanatory models independent of differences in current variability and flow structure. Thus, while we suggest that temperature gradients and broad-scale flow structure are key mechanisms that drive species turnover and community seriation and heterogeneity we cannot exclude the possible influence of additional factors.

## Conclusion

We found strong regional seriation of seaweed communities along the LC and the EAC in temperate Australia. Communities within the LC had lower species turnover and less variation than communities situated within the EAC. These patterns are likely to be predominantly determined by a combination of seaweed temperature tolerances and current-driven dispersal. In conclusion, our findings support the hypothesis that continental-scale currents impart a strong footprint on regional community organisation, and that the coupling of ocean currents and biological structure is a general process that transcends taxa and spatial scales.

## Supporting Information

Table S1 **Details of the 17 marine bioregions used as units of analyses for species turnover among seaweed communities within the Leeuwin Current and East Australia Current.**
(DOCX)

Table S2 **Percent shared species between pairs of all bioregions.**
(DOCX)

## References

[1] Nekola JC, White PS (1999) The Distance Decay of Similarity in Biogeography and Ecology. J Biogeogr 26: 867-878. doi:10.1046/j.1365-2699.1999.00305.x.

[2] Qian H (2009) Beta diversity in relation to dispersal ability for vascular plants in North America. Glob Ecol Biogeogr 18: 327-332. doi:10.1111/j.1466-8238.2009.00450.x.

[3] Hedgpeth JW (1957) Marine Biogeography 1. Geological Society of America pp. 359-382.

[4] Hubbs CL (1948) Changes in the fish fauna of Western North America correlated with the changes in ocean temperature. J Mar Res 379: 459-482.

[5] Wallace AR (1876) The geographical distribution of animals, with a study of the relation of living and extinct faunas as elucidating the past changes of the earth’s surface. London: Macmillan and Co.

[6] von Humboldt A, Bonpland A (1805) Essai sur la géographie des plantes: accompagné d'un tableau physique des régions équinoxiales, fondé sur des mesures exécutées, depuis le dixième degré de latitude boréale jusqu'au dixième degré de latitude australe, pendant les années 1799, 1800, 1801, 1802 et 1803: Chez Levrault, Schoell et compagnie, libraires..

[7] Schils T, Wilson SC (2006) Temperature threshold as a biogeographic barrier in northern Indian Ocean macroalgae. J Phycol 42: 749-756. doi:10.1111/j.1529-8817.2006.00242.x.

[8] van den Hoek C (1982) Phytogeographic distribution of groups of benthic marine algae in the North Atlantic Ocean. A review of experimental evidence from life history studies. Helgol Meeresunters 35: 153-214. doi:10.1007/BF01997551.

[9] Tittensor DP, Mora C, Jetz W, Lotze HK, Ricard D et al. (2010) Global patterns and predictors of marine biodiversity across taxa. Nature 466: 1098-1101. doi:10.1038/nature09329. PubMed: 20668450. 20668450

[10] Belanger CL, Jablonski D, Roy K, Berke SK, Krug AZ et al. (2012) Global environmental predictors of benthic marine biogeographic structure. Proc Natl Acad Sci USA 109: 14046-14051. doi:10.1073/pnas.1212381109. PubMed: 22904189. 22904189 PMC3435205

[11] Watson JR, Hays CG, Raimondi PT, Mitarai S, Dong C et al. (2011) Currents connecting communities: nearshore community similarity and ocean circulation. Ecology 92: 1193-1200. doi:10.1890/10-1436.1. PubMed: 21797147. 21797147

[12] Coleman MA, Roughan M, Macdonald HS, Connell SD, Gillanders BM et al. (2011) Variation in the strength of continental boundary currents determines continent-wide connectivity in kelp. J Ecol 99: 1026–1032. doi:10.1111/j.1365-2745.2011.01822.x.

[13] Gaylord B, Gaines SD (2000) Temperature or transport? Range limits in marine species mediated solely by flow. Am Nat 155: 769-789. doi:10.1086/303357. PubMed: 10805643. 10805643

[14] Fraser CI, Nikula R, Waters JM (2011) Oceanic rafting by a coastal community. Proc R Soc Lond B Biol Sci, 278: 649–655. PubMed: 20843850. 10.1098/rspb.2010.1117 PMC3030839 20843850

[15] Caley MJ, Carr MH, Hixon MA, Hughes TP, Jones GP et al. (1996) Recruitment and the local dynamics of open marine populations. Annu Rev Ecol Syst 27: 477-500. doi:10.1146/annurev.ecolsys.27.1.477.

[16] Connolly SR, Menge BA, Roughgarden J (2001) A latitudinal gradient in recruitment of intertidal invertebrates in the northeast Pacific Ocean. Ecology 82: 1799–1813. doi:10.1890/0012-9658(2001)082[1799:ALGIRO]2.0.CO;2.

[17] Menge BA, Lubchenco J, Bracken MES, Chan F, Foley MM et al. (2003) Coastal oceanography sets the pace of rocky intertidal community dynamics. Proc Natl Acad Sci U_S_A 100: 12229–12234. doi:10.1073/pnas.1534875100. PubMed: 14512513. 14512513 PMC218741

[18] Broitman BR, Blanchette CA, Gaines SD (2005) Recruitment of intertidal invertebrates and oceanographic variability at Santa Cruz Island, California. Limnol Oceanogr 50: 1473–1479. doi:10.4319/lo.2005.50.5.1473.

[19] Banks SC, Piggots MP, Williamson JE, Bové U, Holbrook NJ et al. (2007) Oceanographic variability and coastal topography shape genetic structure in a long-dispersing sea urchin. Ecology 88: 3055-3064. doi:10.1890/07-0091.1. PubMed: 18229840. 18229840

[20] Buchanan J, Zuccarello GC (2012) Decoupling of short and long distance dispersal pathways in the endemic New Zealand seaweed Carpophyllum maschalocarpum (Phaeophyceae, Fucales). J Phycol 48: 518-529. doi:10.1111/j.1529-8817.2012.01167.x. 27011067

[21] Ridgway KR, Condie SA (2004) The 5500-km-long boundary flow off western and southern Australia. J Geophys Res Oceans 219: C04017.

[22] Ridgway KR, Dunn JR (2003) Mesoscale structure of the mean East Australian Current system and its relationship with topography. Prog Oceanogr 56: 189–222. doi:10.1016/S0079-6611(03)00004-1.

[23] Roughan M, Middleton JH (2004) On the East Australian Current: variability, encroachment, and upwelling. J Geophys Res 109: C07003. doi:10.1029/2003JE002224.

[24] Condie SA, Mansbridge JV, Cahill ML (2011) Contrasting local retention and cross-shore transports of the East Australian Current and the Leeuwin Current and their relative influences on the life histories of small pelagic fishes. Deep Sea Res II Topical Stud Oceanogr 58: 606-615. doi:10.1016/j.dsr2.2010.06.003.

[25] Commonwealth of Australia (2005) National Marine Bioregionalisation of Australia.

[26] AVH (2013) The Council of Heads of Australasian Herbaria, Australia’s Virtual Herbarium. Retrieved onpublished at whilst December year 1111 from http://avh.chah.org.au.

[27] Waters JM, Wernberg T, Connell SD, Thomsen MS, Zuccarello GC et al. (2010) Australia's marine biogeography revisited: Back to the future? Austral Ecol 35: 988-992. doi:10.1111/j.1442-9993.2010.02114.x.

[28] Guiry MD, Guiry GM (2013) AlgaeBase. World-wide electronic publication. National University of Ireland, Galway http://www.algaebase.org .

[29] Shaffer HB, Fisher RN, Davidson C (1998) The role of natural history collections in documenting species declines. Trends Ecol Evol 13: 27-30. doi:10.1016/S0169-5347(97)01177-4. PubMed: 21238186. 21238186

[30] Smale DA, Wernberg T (2013) Extreme climatic event drives range contraction of a habitat-forming species. Proc R Soc Lond B 280: 20122829 PubMed: 23325774. 10.1098/rspb.2012.2829 PMC3574333 23325774

[31] Connell SD, Russell BD, Turner DJ, Shepherd SA, Kildea T et al. (2008) Recovering a lost baseline: missing kelp forests from a metropolitan coast. Mar Ecol Prog S 360: 63-72. doi:10.3354/meps07526.

[32] Phillips JA, Blackshaw JK (2011) Extirpation of Macroalgae (Sargassum spp.) on the Subtropical East Australian Coast. Conserv Biol 25: 913-925. doi:10.1111/j.1523-1739.2011.01727.x. PubMed: 21902718. 21902718

[33] Wernberg T, Russell BD, Thomsen MS, Gurgel CF, Bradshaw CJ et al. (2011) Seaweed Communities in Retreat from Ocean Warming. Curr Biol 21: 1828-1832. doi:10.1016/j.cub.2011.09.028. PubMed: 22036178. 22036178

[34] Anderson MJ, Gorley RN, Clarke KR (2008) PERMANOVA+ for PRIMER: Guide to software and statistical methods. Plymouth, UK: PRIMER-E Ltd. p. 214.

[35] Clarke KR, Gorley RN (2006) Primer v6: user manual/tutorial. Plymouth, UK: PRIMER-E Ltd. p. 190.

[36] Wernberg T, Russell BD, Moore PJ, Ling SD, Smale DA et al. (2011) Impacts of climate change in a global hotspot for temperate marine biodiversity and ocean warming. J Exp Mar Biol Ecol 400.

[37] Qian H, Ricklefs RE (2007) A latitudinal gradient in large-scale beta diversity for vascular plants in North America. Ecol Lett 10: 737-744. doi:10.1111/j.1461-0248.2007.01066.x. PubMed: 17594429. 17594429

[38] Virola T, Kaitala V, Lammi A, Siikamaeki P, Suhonen JA (2001) Geographical patterns of species turnover in aquatic plant communities. Freshw Biol 46: 1471-1478. doi:10.1046/j.1365-2427.2001.00767.x.

[39] Qian H, Badgley C, Fox DL (2009) The latitudinal gradient of beta diversity in relation to climate and topography for mammals in North America. Glob Ecol Biogeogr 18: 111-122. doi:10.1111/j.1466-8238.2008.00415.x.

[40] Clarke A, Lidgard S (2000) Spatial patterns of diversity in the sea: bryozoan species richness in the North Atlantic. J Anim Ecol 69: 799-814. doi:10.1046/j.1365-2656.2000.00440.x. 29313988

[41] Waters JM (2008) Marine biogeographical disjunction in temperate Australia: historical landbridge, contemporary currents, or both? Divers Distrib 14: 692-700. doi:10.1111/j.1472-4642.2008.00481.x.

[42] Fraser CI, Spencer HG, Waters JM (2009) Glacial oceanographic contrasts explain phylogeography of Australian bull kelp. Mol Ecol (. (2009)) PubMed: 19389161. 10.1111/j.1365-294X.2009.04201.x 19389161

[43] Lüning K (1990) Seaweeds: their environment, biogeography and ecophysiology. Chichester: John Wiley & Sons p. 600.

[44] Breeman AM (1988) Relative importance of temperature and other factors in determining geographic boundaries of seaweeds: experimental and phenological evidence. Helgol Meeresunters 42: 199-241. doi:10.1007/BF02366043.

[45] Womersley HBS (1990) Biogeography of Australasian Marine Macroalgae. In: Clayton MN King RJ Biology of Marine Plants. Melbourne: Longman Chesire. pp. 367-381

[46] Adey WH, Steneck RS (2001) Thermogeography over time creates biogeographic regions: a temperature/space/time-integrated model and an abundance-weighted test for benthic marine algae. J Phycol 37: 677-698. doi:10.1046/j.1529-8817.2001.00176.x.

[47] Pielou EC (1977) The latitudinal spans of seaweed species and their patterns of overlap. J Biogeogr 4: 299-311. doi:10.2307/3038189.

[48] Pielou EC (1978) Latitudinal overlap of seaweed species: evidence for quasi-sympatric speciation. J Biogeogr 5: 227-238. doi:10.2307/3038038.

[49] Roy K, Jablonski D, Valentine JW, Rosenberg G (1998) Marine latitudinal diversity gradients: tests of causal hypotheses. Procedings of the National Academy of Science USA 45 pp. 3699-3702. PubMed: 9520429. 10.1073/pnas.95.7.3699 PMC19899 9520429

[50] Clarke A (1993) Temperature and extinction in the sea; a physiologist's view. Paleobiology 19: 499-518.

[51] O'Hara TD, Poore GCB (2000) Patterns of distribution for southern Australian marine echinoderms and decapods. J Biogeogr 27: 1321-1335. doi:10.1046/j.1365-2699.2000.00499.x.

[52] Smale DA, Kendrick GA, Wernberg T (2010) Assemblage turnover and taxonomic sufficiency of subtidal macroalgae at multiple spatial scales. J Exp Mar Biol Ecol 384: 76-86. doi:10.1016/j.jembe.2009.11.013.

[53] Condie SA, Waring J, Mansbridge JV, Cahill ML (2005) Marine connectivity patterns around the Australian continent. Environ Model Softw 20: 1149-1157. doi:10.1016/j.envsoft.2004.07.005.

[54] van den Hoek C (1987) The possible significance of long-range dispersal for the biogeography of seaweeds. Helgol Meeresunters 41: 261-272. doi:10.1007/BF02366191.

[55] Nikula R, Fraser CI, Spencer HG, Waters JM (2010) Circumpolar dispersal by rafting in two subantarctic kelp-dwelling crustaceans. Mar Ecol Prog S 405: 221-230. doi:10.3354/meps08523.

[56] Kendrick GA, Walker DI (1991) Dispersal distances for propagules of *Sargassum* *spinuligerum* (Sargassaceae, Phaeophyta) measured directly by vital staining and venturi suction sampling. Mar Ecol Prog S 79: 133-138. doi:10.3354/meps079133.

[57] Norton TA (1980) Sink, swim or stick: The fate of Sargassum muticum propagules. Br Phycol J 15: 197-198.

[58] Norton TA (1992) Dispersal by macroalgae. Br Phycol J 27: 293-301. doi:10.1080/00071619200650271.

[59] Ayers KL, Waters JM (2005) Marine biogeographic disjunction in central New Zealand. Mar Biol 147: 1045-1052. doi:10.1007/s00227-005-1632-7.

[60] Connell SD, Irving AD (2008) Integrating ecology with biogeography using landscape characteristics: a case study of subtidal habitat across continental Australia. J Biogeogr 35: 1608-1621. doi:10.1111/j.1365-2699.2008.01903.x.

[61] Wernberg T, Thomsen MS, Tuya F, Kendrick GA, Staehr PA et al. (2010) Decreasing resilience of kelp beds along a latitudinal temperature gradient: potential implications for a warmer future. Ecol Lett 13: 685-694. doi:10.1111/j.1461-0248.2010.01466.x. PubMed: 20412279. 20412279

[62] Stæhr PA, Wernberg T (2009) Physiological responses of *Ecklonia* *radiata* (Laminariales) to a latitudinal gradient in ocean temperature. J Phycol 45: 91-99. doi:10.1111/j.1529-8817.2008.00635.x. 27033648

[63] Russell BD, Thompson J-AI, Falkenberg LJ, Connell SD (2009) Synergistic effects of climate change and local stressors: CO2 and nutrient-driven change in subtidal rocky habitats. Glob Change Biol 15: 2153-2162. doi:10.1111/j.1365-2486.2009.01886.x.

[64] Connell SD, Russell BD (2010) The direct effects of increasing CO2 and temperature on non-calcifying organisms: increasing the potential for phase shifts in kelp forests. Proceedings of the Royal Society B: Biological Sciences. PubMed: 20053651 10.1098/rspb.2009.2069 PMC2871943 20053651

[65] Phillips J (2001) Marine macroalgal biodiversity hotspots: why is there high species richness and endemism in southern Australian marine benthic flora? Biodivers Conserv 10: 1555-1577. doi:10.1023/A:1011813627613.

[66] Airoldi L, Beck MW (2007) Loss, status and trends for coastal marine habitats of Europe. Oceanogr Mar Biol Annu Rev 35: 345-405.

[67] Gorman D, Russell BD, Connell SD (2009) Land-to-sea connectivity: linking human-derived terrestrial subsidies to subtidal habitat change on open rocky coasts. Ecol Appl 19: 1114-1126. doi:10.1890/08-0831.1. PubMed: 19688920. 19688920

[68] Suthers IM, Waite AM (2007) Coastal oceanography and ecology. in Marine Ecology (edi. South Dakota: Connell, Gillanders, BM); Oxford University Press 199-226.

[69] O'Hara T (2000) Patterns of temperate marine species diversity at the continental scale. East Melbourne, Australia chapter 3. Department of Natural Resources and Environment.

