# Supplementary material for: The Footprint of Continental-Scale Ocean Currents on the Biogeography of Seaweeds
Source: PLoS One. 2013 Nov 8;8(11):e80168. doi: 10.1371/journal.pone.0080168 (PMC3832649; doi:10.1371/journal.pone.0080168)
Supplement: Table S1 — Details of the 17 marine bioregions used as units of analyses for species turnover among seaweed communities within the Leeuwin Current and East Australia Current. (DOCX) [file pone.0080168.s001.docx]

**Table S1.** Details of the 17 marine bioregions used as units of analyses for the biogeographic comparisons of species turnover within the Leeuwin Current (LC) and the East Australia Current (EAC) systems. Regions were based on the National Marine Bioregionalistation of Australia [[25](#_ENREF_25)] (see also Fig. 1). Coastal distances were estimated from measurements using a flexible ruler on maps of the bioregions [[25](#_ENREF_25)]. Herbarium records indicate the number of unique records downloaded from Australia’s Virtual Herbarium ([www.sapac.edu.au/avh/](http://www.sapac.edu.au/avh/)) from each region, and species richness is the number of unique species identified from those records, in each region.

| **Bioregion number** | **Current system** | **IMCRA**  **Region** | **Coastal extent (km)** | **Herbarium**  **records** | **Species**  **richness** |
| --- | --- | --- | --- | --- | --- |
| 1 | LC | Central West Coast | 547 | 3187 | 455 |
| 2 | LC | Leeuwin-Naturaliste | 497 | 6528 | 621 |
| 3 | LC | WA South coast | 1024 | 2184 | 449 |
| 4 | LC | Eucla | 1045 | 681 | 270 |
| 5 | LC | Murat | 290 | 1607 | 374 |
| 6 | LC | Eyre | 1105 | 13079 | 808 |
| 7 | LC | Spencer, North Spencer and St Vincent Gulfs | 1221 | 11875 | 835 |
| 8 | LC | Coorong | 248 | 4571 | 606 |
| 9 | LC | Otway | 736 | 8038 | 747 |
| 10 | LC | Victorian Embayments, Central Victoria, Central Bass Strait, Boags | 1281 | 15794 | 869 |
| 11 | EAC | Bruny | 532 | 3899 | 523 |
| 12 | EAC | Freycinet | 307 | 1384 | 353 |
| 13 | EAC | Flinders | 484 | 584 | 214 |
| 14 | EAC | Twofold Shelf | 501 | 1454 | 360 |
| 15 | EAC | Batemans Shelf | 267 | 2135 | 323 |
| 16 | EAC | Hawkesbury Shelf | 331 | 2724 | 380 |
| 17 | EAC | Manning Shelf | 281 | 464 | 148 |
