# Supplementary material for: The Footprint of Continental-Scale Ocean Currents on the Biogeography of Seaweeds
Source: PLoS One. 2013 Nov 8;8(11):e80168. doi: 10.1371/journal.pone.0080168 (PMC3832649; doi:10.1371/journal.pone.0080168)
Supplement: Table S2 — Percent shared species between pairs of all bioregions. (DOCX) [file pone.0080168.s002.docx]

**Table S2.** Percent shared species of the combined species pool for all pair-wise combinations of bioregions. Green region pairs are from within the Leeuwin Current, white region pairs are between currents comparisons and blue region pairs are from within the East Australia Current. Region numbers (white on black) refer to Fig 1.

| **1** | **2** | **3** | **4** | **5** | **6** | **7** | **8** | **9** | **10** | **11** | **12** | **13** | **14** | **15** | **16** | **17** |
| --- | --- | --- | --- | --- | --- | --- | --- | --- | --- | --- | --- | --- | --- | --- | --- | --- |
| **1** | 36 | 30 | 24 | 26 | 25 | 25 | 24 | 22 | 21 | 16 | 14 | 16 | 15 | 13 | 15 | 8 |
|  | **2** | 33 | 22 | 27 | 30 | 29 | 27 | 26 | 26 | 18 | 16 | 17 | 17 | 16 | 18 | 9 |
|  |  | **3** | 26 | 29 | 29 | 29 | 28 | 25 | 25 | 19 | 17 | 20 | 17 | 15 | 17 | 8 |
|  |  |  | **4** | 29 | 23 | 22 | 25 | 20 | 18 | 15 | 16 | 20 | 15 | 11 | 11 | 7 |
|  |  |  |  | **5** | 29 | 28 | 29 | 25 | 24 | 20 | 19 | 21 | 19 | 13 | 15 | 8 |
|  |  |  |  |  | **6** | 41 | 37 | 37 | 37 | 28 | 23 | 18 | 21 | 15 | 16 | 7 |
|  |  |  |  |  |  | **7** | 36 | 37 | 38 | 28 | 23 | 18 | 22 | 16 | 19 | 8 |
|  |  |  |  |  |  |  | **8** | 37 | 34 | 29 | 25 | 22 | 24 | 16 | 17 | 8 |
|  |  |  |  |  |  |  |  | **9** | 38 | 31 | 27 | 20 | 23 | 16 | 18 | 8 |
|  |  |  |  |  |  |  |  |  | **10** | 33 | 27 | 19 | 23 | 17 | 18 | 9 |
|  |  |  |  |  |  |  |  |  |  | **11** | 33 | 20 | 24 | 17 | 17 | 8 |
|  |  |  |  |  |  |  |  |  |  |  | **12** | 23 | 25 | 16 | 16 | 8 |
|  |  |  |  |  |  |  |  |  |  |  |  | **13** | 21 | 25 | 31 | 21 |
|  |  |  |  |  |  |  |  |  |  |  |  |  | **14** | 25 | 21 | 13 |
|  |  |  |  |  |  |  |  |  |  |  |  |  |  | **15** | 31 | 23 |
|  |  |  |  |  |  |  |  |  |  |  |  |  |  |  | **16** | 20 |
|  |  |  |  |  |  |  |  |  |  |  |  |  |  |  |  | **17** |
